# Potentiation of Gelonin Cytotoxicity by Pulsed Electric Fields

**DOI:** 10.3390/ijms26020458

**Published:** 2025-01-08

**Authors:** Olga N. Pakhomova, Eleni Zivla, Giedre Silkuniene, Mantas Silkunas, Andrei G. Pakhomov

**Affiliations:** Frank Reidy Research Center for Bioelectrics, Old Dominion University, Norfolk, VA 23508, USA; opakhomo@odu.edu (O.N.P.); gsilkuni@odu.edu (G.S.); msilkuna@odu.edu (M.S.)

**Keywords:** electroporation, electropermeabilization, electrochemotherapy, cancer ablation, irreversible electroporation, pulsed field ablation

## Abstract

Gelonin is a ribosome-inactivating protein with extreme intracellular toxicity but poor permeation into cells. Targeted disruption of cell membranes to facilitate gelonin entry is explored for cancer and tissue ablation. We demonstrate a hundreds- to thousands-fold enhancement of gelonin cytotoxicity by pulsed electric fields in the T24, U-87, and CT26 cell lines. The effective gelonin concentration to kill 50% of cells (EC_50_) after electroporation ranged from <1 nM to about 100 nM. For intact cells, the EC_50_ was unattainable even at the highest gelonin concentration of 1000 nM, which reduced cell survival by only 5–15%. For isoeffective electroporation treatments using 300 ns, 9 µs, and 100 µs pulses, longer pulses were more efficient at lowering gelonin EC_50_. Increasing the electric field strength of 8, 100 µs pulses from 0.65 to 1.25 kV/cm reduced gelonin EC_50_ from 128 nM to 0.72 nM. Conversely, the presence of 100 nM gelonin enabled a more than 20-fold reduction in the number of pulses required for equivalent cell killing. Pulsed electric field-mediated delivery of gelonin shows promise for hyperplasia ablation at concentrations sufficiently low to minimize or avoid systemic toxicity.

## 1. Introduction

Gelonin is a plant-derived type I ribosome-inactivating protein (RIP). Like all RIPs, it exhibits strong cytotoxic effects by inactivating ribosomes and blocking protein synthesis. RIPs exert their cytotoxic effects enzymatically, meaning that only a small number of toxin molecules need to reach the cytoplasm to induce a potent cytotoxic response [1,2,3,4]. However, the ability of type I RIPs to reach the cytoplasm is limited. They consist of an enzymatic domain only and lack the lectin domain, which facilitates cell surface attachment and intracellular penetration of type II RIPs such as ricin. Type I RIPs may be admitted into cells by different types of endocytosis, followed by their accumulation and degradation in late endosomes and lysosomes. Their escape from lysosomal breakdown is rare, resulting in low cytotoxicity [4,5,6,7].

A combination of high intracellular toxicity with poor permeation into cells has made type I RIPs attractive to build molecular constructs that target and kill cancer cells while having low systemic toxicity. Type I RIP-based targeting toxins have been under development as cancer therapies for over three decades. In these constructs, a targeting domain such as cancer-specific antibodies, growth factors, cytokines, tumor-homing peptide, or cell-penetrating peptides, is coupled to toxin cargo [1,2,3,7,8,9,10]. In animal and human trials, targeted toxins incorporating plant-derived type 1 RIPs showed efficacy against a range of hematologic malignancies [1,2,3]; bladder carcinoma [11]; hepatocellular carcinoma [12]; and renal, ovary, breast, gastric, pancreas, non-small cell lung and colorectal cancers [1,13]. Two RIP-based drugs derived from bacterial toxins have been approved by the FDA for the treatment of hematologic malignancies [14,15].

Gelonin linked to the CD33 antibody was evaluated against myeloid leukemia in phase I human trials [14,16,17]. It showed anti-leukemia activity in just 26% of patients at the maximum tolerable dose of 28 mg/m^2^. Progress has been hindered by the limited availability of the conjugates and an insufficient therapeutic window between effective anti-cancer doses and toxic effects.

An alternative or a supplement to chemical targeting of RIP toxins to solid tumors could be a physical disruption of cell membranes, e.g., by sonoporation or electroporation. Applying strong pulsed electric fields locally across a tumor can open the intracellular access to chemotherapeutic drugs with a low capacity to cross the intact cell membrane. This method, presently known as electrochemotherapy, has been introduced over three decades ago [18] and progressed into an established, well-recognized treatment option [19,20,21,22,23]. More than 40 types of tumors have been shown to respond to electrochemotherapy, including those incurable with standard chemotherapy and not suitable for excision surgery. Nearly all clinical and veterinary applications of electrochemotherapy rely on just two cytotoxic drugs, bleomycin and cisplatin, which enable a significant safety margin between effective doses and adverse off-site effects. Electroporation reduced the effective concentration to achieve 50% cell killing (EC_50_) 100–5000 times for bleomycin and up to 12.5 times for cisplatin [24,25]. Dozens of other cytotoxic compounds have been tested for enhanced cancer cell killing when combined with electroporation, but most showed little or no potentiation [25,26,27].

Bleomycin and cisplatin kill cells by inflicting DNA damage, thereby targeting predominantly the actively dividing cells, whether cancer or not. Although most tumors respond to electrochemotherapy, its efficiency varies widely [23,27]. For example, in head and neck cancer, the overall response rate ranged from 0 to 100%, while the complete response rate ranged between 0 and 83.3% [23]. The identification of new drugs whose toxicity relies on a qualitatively different mechanism and is potentiated by electroporation could expand the toolbox and the spectrum of electrochemotherapy. Gelonin targets protein synthesis, and this distinct mechanism may provide therapeutic advantages in tumors or tissues that respond poorly to bleomycin and cisplatin.

This study examined the potentiation of gelonin cytotoxicity by pulsed electric fields in three cancer cell lines. We established that gelonin EC_50_ in electroporated cells reaches into the sub-nanomolar range, that is more than 1000-fold below the EC_50_ in intact cells. To the extent studied, increasing the electric pulse duration and the electric field strength synergistically potentiated gelonin cytotoxicity.

## 2. Results

### 2.1. Electroporation Assists Cell Killing by Gelonin

The experiments were performed in CT26 mouse colon carcinoma fibroblast cells, T24 human urinary bladder carcinoma epithelial cells, and U-87 human glioblastoma epithelial cells. They were suspended in the respective growth media with gelonin at concentrations from 0 (vehicle control) to 1000 nM and treated by pulsed electric fields in electroporation cuvettes. A pulse generator was set to deliver either a 10 Hz train of five 9-µs pulses at 2 kV/cm or no pulses (sham-exposed control). These parameters were determined in preliminary experiments as the dose that just marginally reduced cell viability, by 10–15%. Viability was measured at 24 h after pulse treatments by the conversion rate of resazurin to resorufin (Presto Blue assay). Viability measured in sham-exposed vehicle control samples was taken as a 100% reference. Viability measurements with metabolic assays like Presto Blue are generally attributed to changes in cell survival and metabolism but may also be influenced by the enzymatic activity of cell debris.

**Figure 1:**
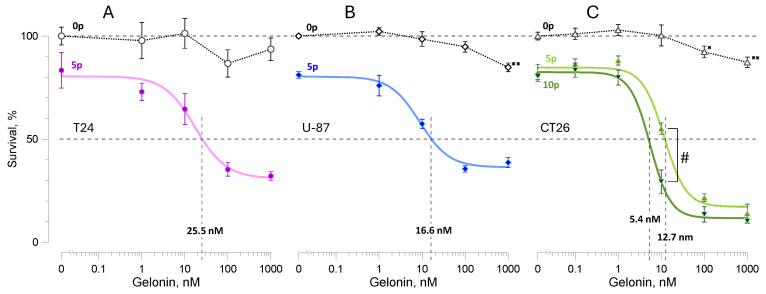
Potentiation of gelonin cytotoxicity by pulsed electric fields in T24 (**A**), U-87 (**B**), and CT26 (**C**) cell lines. Viability was measured 24 h after electroporation with 9-µs, 2 kV/cm pulses at 10 Hz. Samples were treated with 5 pulses (“5p”, **A**–**C**) or 10 pulses (“10p”, **C**) in the presence of gelonin at 0.1–1000 nM or the vehicle (0 nM). Control samples (open symbols) underwent the same treatment, but no electric pulses were delivered (sham exposure, “0p”). Viability in the 0p group at 0 nM gelonin was used as the 100% reference. Solid color lines are best fits for the 5p and 10p groups using the Hill equation. The effective gelonin concentrations that reduced viability to 50% (EC_50_) were measured from Hill fits and are shown by vertical dashed lines and legends above the x-axis. Mean ± s.e., *n* = 4–7. * *p* < 0.05, ** *p* < 0.01 for the difference of sham-exposed controls from 100% (one sample, 2-tailed *t*-test). # *p* < 0.01 for the difference between the 5p and 10p groups (unpaired 2-tailed *t*-test). Differences in viability of the electroporated and sham-exposed cells are significant at *p* < 0.05 or better for all gelonin concentrations (not labeled in the graphs).

In all cell lines, the incubation with gelonin without electroporation caused no or just modest viability reduction (Figure 1). In U-87 and CT26 cells, 1000 nM gelonin reduced viability to 85 ± 2% and 87 ± 2.5%, respectively (*p* < 0.01 for a difference from 100%, 2-tailed one sample *t*-test). A lower concentration of 100 nM gelonin reduced the viability of CT26 to 92 ± 2% (*p* < 0.05), with no significant effect in the other cell lines or at the lower concentrations.

Electroporation in the absence of gelonin or when its concentration was at or below 1 nM reduced cell viability to 80–85%. A further increase in gelonin concentration caused a sharp viability reduction followed by a residual resistance plateau at about 30% (T24 and U-87) or 20% (CT26). The dependence of viability on gelonin concentration in electroporated cells had a classic S-shape appearance and could be fit with the Hill equation (correlation coefficients 0.81 in T24 and 0.95 in both U-87 and CT26 cells; *p* < 0.0001 for all cells). The cytotoxic efficiency of the combined treatment was quantified by the effective gelonin concentration that reduced viability to 50% (EC_50_). In samples electroporated with 5 pulses, the EC_50_ values measured from the fits equaled 25.5 nM, 16.6 nM, and 12.7 nM for the T24, U-87, and CT26 cells, respectively. These values could not be accurately compared to EC_50_ values of gelonin in intact cells because even its highest concentration reduced viability by just 10–15%. Instead, a conservative estimate (comparing EC_50_ for the combined treatment with EC_90_ for gelonin only) indicates at least a 100-fold potentiation of gelonin toxicity by electroporation.

More detailed studies focused on CT26 cells, which showed the highest gelonin sensitivity and relatively small residual resistance (Figure 1C). Increasing the electroporation dose to 10 pulses had just a modest effect on viability per se but strongly potentiated the toxicity of gelonin. With 10 nM gelonin, the viability dropped from 65.5 ± 3.2% to 29.7 ± 5.3% (*p* < 0.01, 2-tailed *t*-test) and the EC_50_ decreased to 5.4 nM.

**Figure 2:**
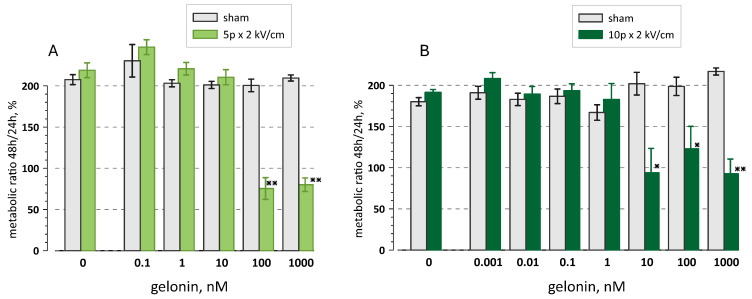
The effect of gelonin and electroporation on the viability change from 24 to 48 h in CT26 cells. Samples were treated with 5 (**A**) or 10 (**B**) electric pulses (9 µs, 2 kV/cm) or were sham-exposed in the presence of different gelonin concentrations (0.001–1000 nM) or vehicle (0 nM). Viability measured at 24 h in each individual sample was taken as 100%. Mean ± s.e., *n* = 4; * *p* < 0.05, ** *p* < 0.01 for the difference from the matching sham-exposed control group (unpaired 2-tailed *t*-test). See Figure 1 and the text for more detail.

To test if the residual resistance at 100 and 1000 nM gelonin truly reflects the metabolic activity of surviving cells, we compared viability at 24 h and 48 h (Figure 2). The measurements at 24 h presented in Figure 1C were used as a 100% reference. By 48 h, the viability doubled in all intact (non-electroporated) samples regardless of gelonin concentration, indicating normal growth of the cell population. Viability also doubled in electroporated cells incubated at gelonin concentrations below EC_50_. However, measurements in electroporated samples with gelonin at 100 and 1000 nM (5 pulses) and at 10–1000 nM (10 pulses) did not increase or even diminished by 48 h. This result suggests that there were no viable cells in these samples already at 24 h and the “residual resistance” readings could be from the enzymatic activity of cell debris. It is also possible that the 24 h measurements were from a mixed population of still dying and viable cells; then, continued destruction of the former and proliferation of the latter could offset each other, yielding 48 h readings still around 100%. However, this balance is expected to shift towards lower readings at higher gelonin concentrations and higher pulse numbers. The lack of any such trend in the data in Figure 2 makes the second interpretation less likely.

### 2.2. Longer Pulses Are More Efficient in Potentiating Gelonin Cytotoxicity

Shorter electric pulses expectedly require stronger electric fields and larger pulse numbers to electroporate cell membrane and influence viability. When these parameters are tuned to affect the viability similarly, shorter nanosecond-range pulses open smaller membrane pores but in larger quantities than longer micro- and millisecond pulses [28]. Gelonin is a relatively large molecule, and it is not known if it can diffuse through electropores or requires a more complex machinery or endocytosis to enter cells [29,30,31]. If it is admitted through the pores, longer pulses which open larger pores could be more efficient at potentiating gelonin cytotoxicity.

This hypothesis was validated by comparing the effects of 300 ns, 9 µs, and 100 µs pulses. In preliminary experiments without gelonin, the electric field strength and pulse numbers were tuned to the isoeffective reduction in viability by pulses of different durations. The protocol of 8, 100-µs pulses was specifically selected to match the standard operating procedures for electrochemotherapy and facilitate future comparisons with the established treatments [19,32,33].

**Figure 3:**
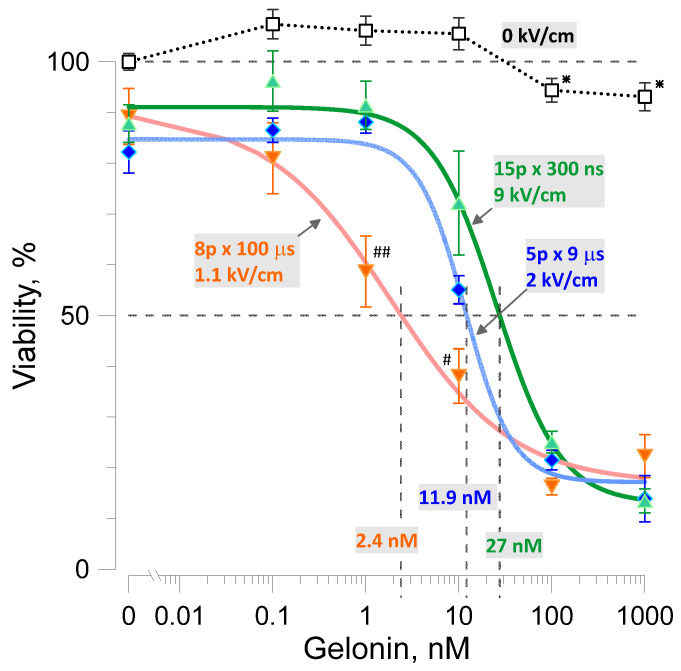
Effect of the electric pulse duration on the potentiation of gelonin cytotoxicity in CT26 cells. Doses of 300-ns, 9-µs, and 100-µs pulses (legends) were adjusted to cause a similar reduction in viability (to 80–90%) in the absence of gelonin. Gelonin EC_50_ values (legends above x-axis) were smaller in cells electroporated by longer electric pulses. Mean ± s.e., *n* = 4 for electric pulse-treated groups. Data for sham-exposed controls (open symbols, 0 kV/cm) were pooled together for all pulse durations, *n* = 12. # *p* < 0.05 and ## *p* < 0.01 for the difference between the samples treated with 100 µs pulses from those treated with either 9 µs or 300 ns pulses (unpaired 2-tailed *t*-test with Dunnett’s correction). * *p* < 0.05 for the difference of sham-exposed controls from 100% (one sample, 2-tailed *t*-test). See Figure 1 and the text for other details.

Without gelonin, trains of 15, 300-ns pulses at 9 kV/cm; trains of 5, 9-µs pulses at 2 kV/cm; and trains of 8, 100-µs pulses at 1.1 kV/cm all reduced cell viability to 80–90% (Figure 3). However, 100-µs pulses were the most efficient at reducing cell viability in the presence of 1 or 10 nM of gelonin (*p* < 0.01 and *p* < 0.05, respectively; two-tailed *t*-test with Dunnet correction). Gelonin EC_50_ was also the lowest at 2.4 nM for 100-µs pulses, versus 11.9 and 27 nM for 9-µs and 300-ns pulses, respectively.

### 2.3. Synergistic Effect of the Electric Field Strength and Gelonin

**Figure 4:**
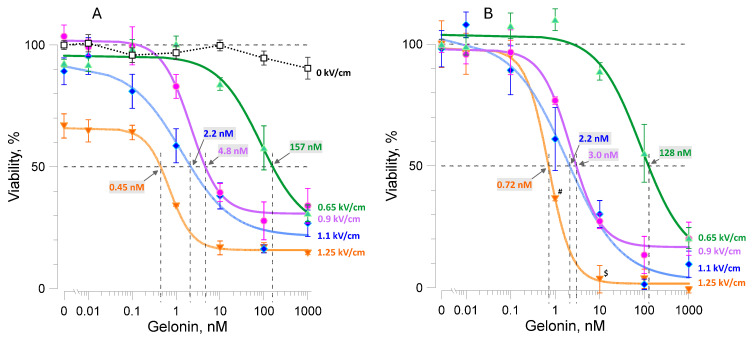
The effect of the electric field strength on the potentiation of gelonin cytotoxicity. (**A**) Viability measured 24 h after exposure to 8, 100-µs pulses at the indicated electric field strengths (kV/cm) in the presence of gelonin at 0.1–1000 nM or the vehicle (0 nM). Control samples (open symbols) were sham exposed (0 kV/cm). EC_50_ values for gelonin (nM) are labeled at the intersections of the Hill fits (solid lines) with a grid line at 50%. (**B**) The same data re-scaled between the viability of electroporated cells without gelonin (100%) and the observed viability minimum (0%). Mean ± s.e., *n* = 4 for pulse-treated groups and *n* = 16 for the pooled sham-exposed group. # *p* < 0.01 for the difference of the 1.25 kV/cm group from the 0.9 and 0.65 kV/cm groups; $ *p* < 0.05 for the difference of the 1.25 kV/cm group from all other groups (unpaired 2-tailed *t*-test with Dunnett’s correction). See Figure 1 and the text for other details.

Trains of 8, 100-µs pulses at 0.65, 0.9, and 1.1 kV/cm had a small or no impact of their own on cell viability but potentiated gelonin cytotoxicity proportionally to the electric field strength (Figure 4A). As the electric field strength increased, the minimal gelonin concentration that significantly reduced viability (*p* < 0.05 compared to 0 kV/cm control) diminished from 10 to 1 and 0.1 nM, respectively, and the EC_50_ lowered from 157 to 4.8 and 2.2 nM. A still stronger electric field of 1.25 kV/cm reduced viability to 67 ± 4% already without gelonin. Viability reached the minimum of about 15% with 10 nM gelonin and stayed at this level despite increasing the drug concentration to 100 and 1000 nM.

The Hill fit of viability data in cells electroporated at 1.25 kV/cm crossed the 50% viability line at 0.45 nM gelonin. However, this value would be misleading as a gelonin EC_50_ because of the substantial reduction in viability by electroporation itself. To isolate the gelonin toxicity, the data were re-calculated using the viability of electroporated cells at 0 nM gelonin as a 100% reference, and the lowest observed viability of 15% was assumed as a zero viability reference for all the groups (see also Section 2.1). The resulting Hill plots (Figure 4B) are the best approximations of gelonin cytotoxicity in cells at different electric field strengths. Gelonin became more toxic in more severely electroporated cells. For example, 1 nM gelonin reduced viability to 37 ± 0.6% following electroporation at 1.25 kV/cm, compared to 61 ± 13% after 1.1 kV/cm and 77 ± 11.4% after 0.9 kV/cm (*p* < 0.01, 2-tailed *t*-test with Dunnet’s correction), and it had no toxicity after the 0.65 kV/cm exposure. Likewise, 10 nM gelonin reduced viability to 3.9 ± 5.2% after electroporation at 1.25 kV/cm, which was significantly (*p* < 0.05) lower than 30.3 ± 5.6%, 27.2 ± 3.2, and 89 ± 4% after the 1.1, 0.9, and 0.65 kV/cm treatments, respectively. The IC_50_ followed the same trend, ranging from 0.72 nM for the most electroporated cells to 128 nM for the least injured ones.

### 2.4. Gelonin Potentiates Cell Inactivation by Pulsed Electric Fields

**Figure 5:**
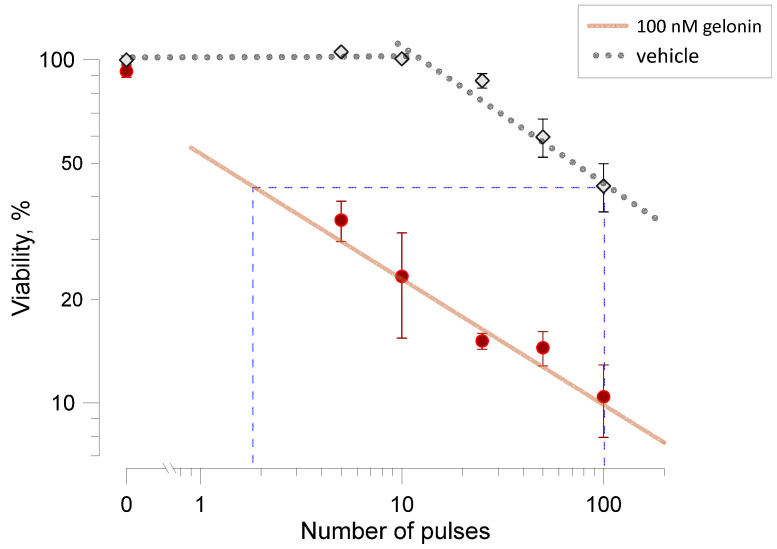
Gelonin potentiates cell inactivation by electroporation. Viability of CT26 cells was measured 24 h after exposure to different numbers of 9-µs, 2 kV/cm pulses, in the presence or absence of 100 nM gelonin (filled and open symbols, respectively). Viability in sham-exposed samples (0 pulses) without the drug was taken as 100%. Solid and dotted lines are the respective best fits of data using the power function; the initial plateau in the no-drug group was excluded from the fit. Mean ± s.e., *n* = 3–4. The difference in viability with and without gelonin is significant at least at *p* < 0.02 (unpaired 2-tailed *t*-test) for all pulse numbers. Dashed lines illustrate the estimated 50-fold difference in the pulse number to achieve the same viability reduction with and without gelonin.

All preceding experiments tested how electroporation enhances the toxicity of gelonin. Conversely, gelonin may enhance the cytotoxicity of electroporation, reducing the number of pulses needed to achieve comparable reductions in cell viability (Figure 5). In this set of experiments, cell viability was measured in samples exposed to varying numbers of electric pulses (from 5 to 100, 9-µs duration, 2 kV/cm) in the presence or absence of 100 nM gelonin. Viability decreased proportionally to the pulse number in both conditions. Same as in earlier reports [34,35], the rate of decrease followed a power function, except for the initial plateau observed without gelonin.

The presence of gelonin reduced viability approximately 4.5 times, with the difference between two conditions being highly significant (*p* < 0.02 or better for all pulse numbers). Trains of just 5 pulses with gelonin and of 100 pulses without it reduced viability to comparable levels, 34.2 ± 6.6% and 42.7 ± 7.3%, respectively. When extrapolated using power function fits in Figure 5, 100 pulses without gelonin produced the same effect as just 2 pulses with it. Thus, the presence of 100 nM gelonin enabled, by different estimates, a 20- to 50-fold reduction in the number of pulses required to achieve the same decline in cell viability.

## 3. Discussion

The principal conclusion made in this study is profound potentiation of gelonin cytotoxicity by electroporation. Cell killing could be enhanced more than 1000-fold, which is comparable to or even better than that for bleomycin, the agent that is the most widely used in electrochemotherapy. This result encourages further exploration of cell killing synergy between gelonin and electric pulses toward novel ablation therapies, from cell lines to tissues and in vivo studies. It must be paralleled with mechanistic studies of how exactly gelonin gets into cells and escapes endosomal entrapment; its selectivity against different cell types; and cell death pathways and their immunogenicity. While this list is long, the extensive experience with “conventional” electrochemotherapy as well as the already known systemic toxicity of gelonin will facilitate the translation.

In contrast to bleomycin and cisplatin, which both rely on DNA damage to kill cells, gelonin disrupts protein synthesis. This difference may account for a reduced gelonin selectivity against certain types of cancer but may benefit ablation of slow-growing hyperplasia and non-dividing tissues where bleomycin and cisplatin would not work. An example is pulsed field cardiac ablation (PFCA), which is a novel, faster, and presumably safer technique for treating arrhythmias such as atrial fibrillation [36,37]. Unlike traditional methods of thermal ablation, PFCA relies on the cardiac tissue damage by irreversible electroporation. It reduces the risks of off-site injuries but is not free of adverse side effects (e.g., due to microbubble formation). A profound reduction in the number or amplitude of pulses by combining PFCA with a brief gelonin perfusion through coronary arteries could further speed up the procedure and bring it up to the next safety level. Although the benefits of gelonin in PMCA remain to be explored, its potential utility for cardiac ablation highlights the difference from bleomycin and cisplatin, which are not expected to potentiate the removal of non-dividing cardiac cells.

Aside from the medical prospects, the potentiation of gelonin cytotoxicity by electroporation raises questions about the fundamental molecular mechanisms of membrane permeabilization by pulsed electric fields. Gelonin molecule (~29 kDa) may be too big to enter cells by diffusion through electropores. Previous studies postulated that pulses shorter than 1 mS do not permeabilize cells for molecules with molecular mass higher than 10 kDa [31], yet in our experiments, even much shorter 300-ns pulses increased gelonin toxicity more than 100-fold (Figure 3). Multiple studies suggest that electropores open once the induced membrane potential reaches a critical level of 200–300 mV and may grow in diameter for the duration of the pulse. Consistently with this concept, gelonin toxicity was potentiated stronger by 100-µs pulses than by either 9-µs or 300-ns pulses, suggesting that more gelonin entered through larger pores opened by the longest pulses. However, nanosecond-duration pulses open small pores of about 1 nm in diameter [28,38,39,40,41], and the admittance of a 29 kDa solute with an estimated diameter of 4.1 nM is unlikely. Perhaps the incidental expansion of pores from 1 to 4 nm just by thermal fluctuation is possible, but, to our knowledge, such behavior has not been observed in molecular models or experiments [40,42]. Electric pulse-mediated gelonin entry may turn out to be a complex, multi-step process like electro-mediated gene transfer [29,30].

## 4. Materials and Methods

### 4.1. Cell Lines and Growth Media

Mouse colon carcinoma fibroblast cells (CT26.WT, CRL-2638), human urinary bladder carcinoma epithelial cells (T24, HTB-4), and human glioblastoma epithelial cells (U-87 MG, HTB-14) were obtained from the American Type Culture Collection (ATCC, Manassas, VA, USA). The cell lines were maintained in humidified 5% CO_2_ in air at 37 °C. CT26 cells were cultured in RPMI-1640 medium, T24 cells in McCoy’s 5A medium, and U-87 cells in EMEM medium all obtained from Corning Inc. (Corning, NY, USA). All media were supplemented with 10% fetal bovine serum (Atlanta Biologicals, Norcross, GA, USA), 100 I.U./mL penicillin, 0.1 μg/mL streptomycin (Gibco, Gaithersburg, MD, USA), and 15 mM HEPES (Sigma-Adrich, St. Louis, MO, USA).

### 4.2. Preparation of Samples and Experiment Protocols

On the day of the experiment, the cells were detached with 0.05% trypsin-EDTA (Gibco) and centrifuged at 120× *g* for 5 min. The cell pellets were resuspended in the respective growth media at 200,000 cells/mL.

Serial dilutions of gelonin (Enzo Life Sciences, Farmingdale, NY, USA) to 10× its intended concentrations were prepared on the day of the experiments in a standard physiological solution containing (in mM) 140 NaCl, 5.4 KCl, 2 CaCl_2_, 1.5 MgCl_2_, 10 HEPES, and 10 glucose (pH 7.2–7.3, 300–310 mOsm/kg, 16.4 mS/cm). The chemicals were from Sigma-Adrich and Fisher Scientific (Hampton, NH, USA). The dilutions were stored at 4 °C and added to the cell suspension at 1:9 ratio 5 min before the electric pulse or sham exposures.

After gentle mixing, 100-μL aliquots of the cell suspensions with gelonin or vehicle added were transferred into 1-mm electroporation cuvettes (BioSmith, Vandergrift, PA, USA) and exposed to the pulsed electric field or were sham-exposed in a random order.

The cuvettes were then left for 10 min on the bench at room temperature. Afterwards, the suspension in all cuvettes was diluted with 200 μL of the complete growth medium without gelonin and gently mixed. During the next 10 min, 200 μL of the diluted suspension was collected from each cuvette and transferred to a 96-well plate. The plate was centrifuged at 120× *g* for 5 min, from 20 to 25 min after the electric pulse treatment. The supernatant was removed and replaced with 100 μL of fresh complete growth medium in about 25 min after exposure.

Thus, the cells were exposed to the intended gelonin concentration for 5 min before electroporation and 10 min after it. Incubation continued with a 3-fold lower gelonin concentration for an additional 15 min.

The plates were transferred to a 37 °C, 5% CO_2_ incubator until viability measurements were performed.

### 4.3. Pulsed Electric Field Treatments

Nearly rectangular 300-ns and 9-µs pulses from an AVTECH AVOZ-D2-B-ODA generator (AVTECH Electrosystems, Ottawa, ON, Canada) were delivered to the cuvette via a 50- to 10-Ohm transition module (AVOZ-D2-T, AVTECH Electrosystems) modified into a cuvette holder [43]. Rectangular 100-µs pulses were from a custom-made high-voltage, low-output impedance electroporator [44] driven by an S88K stimulator (Grass Instruments Co., Quincy, MA, USA). In all experiments, pulses were delivered at a 10-Hz repetition rate. For different pulse durations (Figure 3), the electric field strength and pulse numbers were tuned to the isoeffective reduction of viability by 10–15% in the absence of gelonin. Pulse shape and amplitude were monitored with a TDS3052B oscilloscope (Tektronix, Wilsonville, OR, USA). The electric field values were calculated as pulse voltage over a 1-mm distance.

### 4.4. Viability Measurements

At 23 h after pulse treatments, 10 μL of the Presto Blue reagent (Thermo Fisher Scientific, Richmond, VA, USA) was added to each well and incubation continued for 1 h at 37 °C. The plates were read with a Synergy 2 microplate reader (BioTEK, Winooski, VT, USA), with ex./em. settings at 530/590 nm. The data were corrected for background fluorescence by subtracting values for the wells that contained culture medium without cells.

For the experiments shown in Figure 2, the medium with Presto Blue was replaced with the fresh medium after the readings, and the plates were returned to the incubator. The Presto Blue reagent was added again at 47 h and viability measurements were taken at 48 h. Viability at 48 h was expressed relative to readings in the same well at 24 h, taken as 100%.

### 4.5. Data Analysis and Statistics

Most experiments were performed at least four times. Electric pulse treatments were randomized with sham exposures, which were performed in exactly the same manner but no electric pulses were triggered. Viability measured in the sham-exposed vehicle control samples was used as a 100% reference. Graphs, prepared with Grapher 16 (Golden Software, Golden, CO, USA), present the mean data values and their standard error. Viability in the electroporated cells incubated with different gelonin concentrations was fitted with a modified Hill equation:

$$V\left(C_{g}\right)=V_{min}+\frac{V_{max}-V_{min}}{{\left.1+\left(\frac{C_{g}}{{EC}_{midpoint}}\right.\right)}^{n}}\;\;\;\;\;,\;$$

where

*V(C_g_)* is the viability at gelonin concentration *C_g_*;

*V_min_* and *V_max_* are the minimum and maximum measured viability values;

*EC_midpoint_* is the gelonin concentration at which viability is halfway between *V_min_* and *V_max_* (if *V_min_* = 0 and *V_max_* =100%, *EC_midpoint_ = EC*_50_);

*n* is the Hill coefficient controlling the steepness of the curve.

In Figure 5, the data were fitted with a power function. All fits were accomplished with Grapher, and the calculated *p*-values of all fits were better than 0.001.

The statistical comparisons utilized appropriate versions of the *t*-test, as indicated in the text and figures, with Dunnett’s correction when several groups were compared.

## 5. Patents

O.N.P and A.G.P. co-authored a pending patent relevant to this study, application number 63/569,928.

## Data Availability

The raw data supporting the conclusions of this article will be made available by the authors on request.
